# Analogous adaptations in speed, impulse and endpoint stiffness when learning a real and virtual insertion task with haptic feedback

**DOI:** 10.1038/s41598-020-79433-5

**Published:** 2020-12-18

**Authors:** Atsushi Takagi, Giovanni De Magistris, Geyun Xiong, Alain Micaelli, Hiroyuki Kambara, Yasuharu Koike, Jonathan Savin, Jacques Marsot, Etienne Burdet

**Affiliations:** 1 NTT Communication Science Laboratories, 3‐1 Morinosato Wakamiya, Atsugi, Kanagawa 243-0198 Japan; 2 Tokyo Institute of Technology, 4259 Nagatsuta-cho, Yokohama, 226-8503 Japan; 3 Imperial College of Science, Technology and Medicine, South Kensington, London, SW7 2AZ UK; 4 Precursory Research for Embryonic Science and Technology (PRESTO), Japan Science and Technology Agency (JST), 4-1-8 Honcho, Kawaguchi, Saitama 332-0012 Japan; 5 CEA, LIST, LSI, Rue de Noetzlin, 91190 Gif-sur-Yvette, France; 6 Institut National de Recherche Et de Sécurité (INRS), Rue du Morvan, CS 60027, 54519 Vandoeuvre-lès-Nancy, France

**Keywords:** Neuroscience, Motor control

## Abstract

Humans have the ability to use a diverse range of handheld tools. Owing to its versatility, a virtual environment with haptic feedback of the force is ideally suited to investigating motor learning during tool use. However, few simulators exist to recreate the dynamic interactions during real tool use, and no study has compared the correlates of motor learning between a real and virtual tooling task. To this end, we compared two groups of participants who either learned to insert a real or virtual tool into a fixture. The trial duration, the movement speed, the force impulse after insertion and the endpoint stiffness magnitude decreased as a function of trials, but they changed at comparable rates in both environments. A ballistic insertion strategy observed in both environments suggests some interdependence when controlling motion and controlling interaction, contradicting a prominent theory of these two control modalities being independent of one another. Our results suggest that the brain learns real and virtual insertion in a comparable manner, thereby supporting the use of a virtual tooling task with haptic feedback to investigate motor learning during tool use.

## Introduction

Humans can be characterized by their use of complex tools. The skillful use of tools, evident since 35,000 years ago^1^, remains critical to many industrial processes. A common process in the assembly line is the insertion task where pliers are used to insert a metallic clip into a fixture^2^. This seemingly simple task demands coordination as the tool must be controlled in both free motion and during interaction. State-of-the-art robotic algorithms are less robust and slower than a human worker at the insertion task due to the nonlinear dynamics of the task^3^. What makes humans so adept at learning and using tools to interact with the environment?

Unlike reaching movements that have been studied extensively for several decades^4^, the control of force and motor learning during interaction is relatively understudied^5–7^. A drawback of investigating motor learning in a real tooling task is the degradation of the materials involved. During insertion, the clip engraves the fixture with repeated use, thereby reducing the friction and the force needed in proceeding insertions. As such, correlates of motor learning e.g., reduced force, are confounded by the changing dynamics of the task in a real environment.

These confounds can be avoided by investigating motor learning of an insertion task in a virtual environment where the dynamics of the tool and the materials are not subject to degradation after repeated use. A virtual environment poses other advantages, such as freedom in manipulating the dimensions of the tool^8^, and the ability to control the dynamics of the interaction with precision and to observe the progress of motor learning. However, it is unclear whether the behavior of participants learning a virtual insertion task is comparable to those learning real insertion. Non-linear dynamics such as the friction between the tool and the environment that lead to snags are difficult to implement in a virtual environment. Consequently, the motor learning of a virtual task may be faster as the dynamics are simpler and more predictable than in a real environment. To our knowledge, only a handful of studies have compared tool use in a real and virtual environment^9–12^. These studies have reported that participants learning a peg-in-hole tooling task take longer^10^ and exert more force in a virtual environment than in a real one^9^, and highlight the importance of providing haptic feedback of the task to reduce the completion time in the virtual environment^11,12^. However, these studies did not show how the completion time and the tangential force changed over trials, and whether these changes occurred at comparable rates in both environments. If the motor learning in a virtual environment is comparable to that in a real environment, this raises the possibility of accelerating the training of novices by having them practice in a virtual environment prior to the real task.

The main purpose of this study is to compare the correlates of motor learning during a real and virtual insertion task, and document the change in their time-series between and within trials. The correlates of motor learning we focused on were the trial duration, the movement speed, the force impulse and the endpoint stiffness magnitude. We first hypothesized that these variables, when normalized for comparisons, would change in a comparable manner in a real and virtual environment. The trial duration may decrease due to an increase in the movement speed during insertion. The force impulse, or the integral of the force over time after insertion, should decrease to minimize effort since it does not contribute to the task. The endpoint stiffness magnitude may decline with time as the central nervous system (CNS) learns the dynamics of the interaction during insertion^13,14^. In the real insertion task, we measured the average muscle activity of the arm as a measure of the endpoint stiffness magnitude^15^. In the virtual insertion task, the power grasp force was measured as an estimate of the arm’s endpoint stiffness magnitude, owing to the positive linear relationship between them^16^. Our second hypothesis is that the force during the real insertion should decline over time as the fixture degrades with repeated use. However, it should remain unchanged in the virtual environment.

## Materials and methods

### Real insertion task

**Figure 1:**
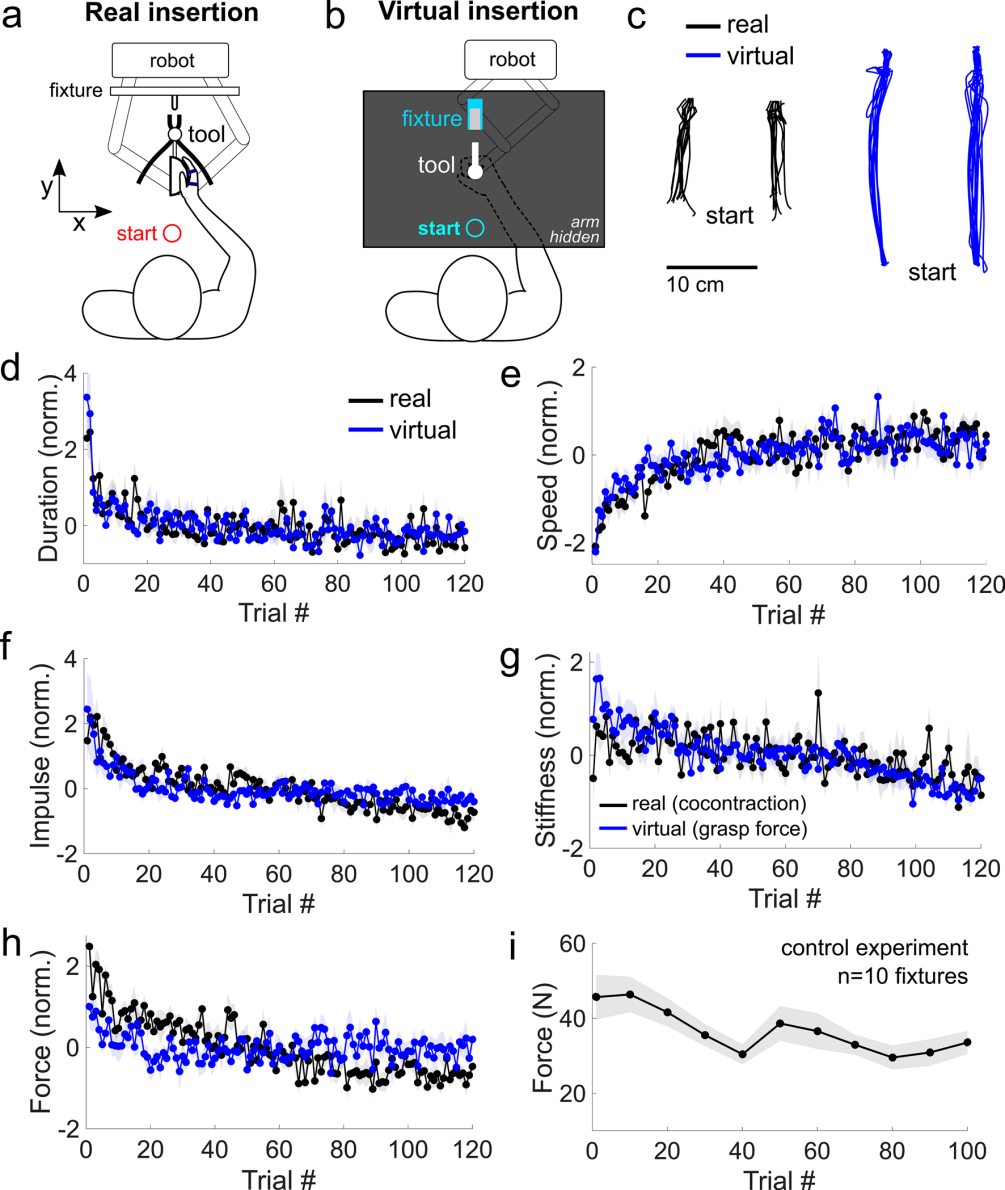
Comparable changes in the correlates of motor learning were observed in the real and virtual insertion task. (**a**) In the real task, the hand was strapped to the end-effector of a robot interface, to which a set of pliers and a metal clip were attached. Participants were instructed to move the hand from the start position and insert the tool into the fixture 120 times. (**b**) A separate group of participants grasped a robotic interface to insert a virtual tool into a fixture. Haptic feedback of the interaction was provided. (**c**) The hand’s trajectory in the early phase from two representative participants in the real (black) and virtual insertion (blue). The hand moved relatively straight from the start to the insertion position. (**d**) Normalized movement duration as a function of trial number in both environments. The duration dropped rapidly in the first few trials, and slowly declined thereafter. (**e**) Normalized speed increased more rapidly in the first half than in the latter half of the task. The change in the speed was comparable between the real and virtual tasks. (**f**) Normalized impulse and (**g**) normalized stiffness, estimated from average normalized muscle activity in the real task and grasp force in the virtual task, both declined as a function of trials in a comparable manner in the real and virtual environments. (**h**) Normalized force during insertion declined in the real insertion task, but not during virtual insertion. (**i**) Control experiment revealed that the force needed to insert the tool with the clip decreased with repeated insertion due to the degradation of the fixture.

The experiment and the procedure were approved by the Imperial College Research Ethics Committee and were performed in accordance with the relevant guidelines and regulations, with all participants providing written informed consent prior to participation. Eight male participants (24 ± 1 years old, all right-handed) were recruited to learn the real insertion task. The participants were seated facing a planar robotic interface^17^. Their right arm was positioned on a horizontal support with an abduction of ≈ 90°, and the right hand with an open palm was strapped to a wrist flange (Fig. 1a). The robotic interface was entirely passive and measured the position, velocity and the force exerted by the hand in the two-dimensional plane at 1 kHz. Participants could see their hand, tool and fixture in the real task. Participants were asked to start their movement from the same position and insert the real tool into the fixture 120 times (Fig. 1a), covering a distance of 10 cm from start to end.

Surface electromyography (EMG) was recorded at 10 kHz from six muscles in the arm: pectoralis major, posterior deltoid, brachii, triceps long-head, flexor carpi radialis and extensor carpi radialis longus from the shoulder, elbow and wrist respectively. To estimate the muscle force, these signals were filtered using a second order high-pass Butterworth filter with a 10 Hz cut-off frequency to remove movement artefacts, then rectified, and finally processed with a second order low-pass Butterworth filter with a 15 Hz cut-off frequency^18^. The filtered EMG was down sampled to 1 kHz for the analysis.

### Virtual insertion task

The experiment and the procedure was approved by the Ethical Review Committee for Epidemiological Studies at the Tokyo Institute of Technology and were performed in accordance with the relevant guidelines and regulations, with all participants providing written informed consent prior to participation. Ten male participants (24 ± 1 years old, all right-handed, none had participated in the real virtual task) were recruited to learn a virtual insertion task. Participants were seated facing the KINARM planar robotic interface from BKIN Technologies^19^ (Fig. 1b). The right arm was positioned on a horizontal support with shoulder abduction of approximately 90°. Participants grasped the handle of the robotic interface with the fingers curled around the cylindrical handle. The arm and the hand were obscured from direct view. A three-axis force sensor (Tec Gihan) was placed between the handle and the palm of the hand to measure the participant’s power grasp force. The participants received visual feedback of the tool’s position (${x}, {y}$) as a rectangular bar with ~ 1:1 scaling on a monitor, such that the movements of the cursor accurately correspond to the movement of the hand. Participants completed 15 point-to-point reaching trials as training prior to undertaking the 120 insertion trials. The movement length was 20 cm. The grasp force and the position, velocity and force in the two-dimensional plane were recorded at 1 kHz.

The virtual tool was 10 cm long with a width of 0.5 cm. The fixture’s inner width was 1 cm. If participants attempted the insertion with the tool’s lateral position $\left|x\right|>0.5$ cm from the center of the fixture, a force

1 $${F}_{y}=4500\left({y}_{1}-y\right).$$

was exerted on the hand, where ${y}_{1}$ is the entrance to the fixture. When the tool is aligned with the fixture such that $\left|x\right|<0.5$ cm, it could be inserted. Inside the fixture, the hand experienced a force of

2 $$F_{y} = \left\{ {\begin{array}{*{20}l} {750\left( {y_{1} - y} \right)} \hfill & {{\text{if}}\;y_{1} < y < y_{2} } \hfill \\ {750\left( {y_{1} - y} \right) + 4500\left( {y_{2} - y} \right)} \hfill & {{\text{if }}y_{2} < y} \hfill \\ 0 \hfill & {{\text{otherwise}}} \hfill \\ \end{array} } \right.$$

where ${y}_{2}$ is the end of the fixture, and a lateral force

3 $$F_{x} = \left\{ {\begin{array}{*{20}l} { - 300x} & {{\text{if}}\;y_{1} < y} \\ 0 & {{\text{otherwise}}} \\ \end{array} } \right.$$

was imposed to prevent the tool from sliding laterally inside the fixture. Even with a large stiffness of 4500 N/m at the end of the fixture, the tool could be inserted beyond this point, giving visual feedback of the tool unnaturally exceeding the fixture. To prevent it from exceeding the fixture’s end ${y}_{2}$, the visual feedback of the tool’s position was capped at $y=\mathrm{min}(y,{y}_{2})$.

### Trial definition and data normalization

The start of each trial was defined as the time when the tangential velocity of the tool i.e., the magnitude of the velocity vector in the *x* and *y* axes, first exceeded 0.02 m/s, and the end of the trial was defined as the time of the peak tangential force during contact inside the fixture. The time taken from the start to the end of each trial is defined as the movement duration. The average tangential speed over the whole trial was defined as the speed. Each quantity was z-normalized for comparisons between participants. As an example, the z-normalized movement duration ${T}^{(\mathrm{N})}$ is given by

4 $${T}^{(\mathrm{N})}=(T-{\mu }_{T})/{\sigma }_{T}$$

where $T$ is the movement duration, ${\mu }_{T}$ is the mean movement duration and ${\sigma }_{T}$ is the standard deviation of the movement duration.

### Force impulse during insertion

Any tangential force exerted after the real or virtual tool was inserted into the fixture i.e., after the peak tangential force, is wasteful. Over trials, the force impulse, defined as the integral of the force after insertion, is expected to decrease due to the minimization of the effort. In both the real and the virtual task, we calculated the impulse from the time of the peak tangential force until the time it returned to zero.

### Estimates of endpoint stiffness magnitude

As muscle activity has an approximately linear contribution to the endpoint stiffness of the arm^15,20^, the magnitude of the endpoint stiffness can be approximated by the mean normalized activity in the arm^21^. In the real task, the filtered activity of each muscle ${m}_{i}$, averaged over a trial, was z-normalized to obtain the normalized activity of each muscle ${m}_{i}^{(\mathrm{N})}$ and then averaged to yield the mean normalized activity

5 $$u^{{\left( {\text{N}} \right)}} = \frac{1}{6}\sum\limits_{i = 1}^{6} {m_{i}^{{\left( {\text{N}} \right)}} } .$$

The normalized activity ${u}^{(\mathrm{N})}$ was taken to be the cocontraction of the arm, and was used as a corollary measure of the arm’s endpoint stiffness magnitude.

We also calculated the normalized cocontraction in each joint by taking the sum of the normalized muscle activity ${m}_{i}^{(\mathrm{N})}$ at the wrist (sum of flexor carpi radialis and extensor carpi radialis longus), the elbow (sum of biceps brachii and triceps long-head) and the shoulder (sum of pectoralis major and posterior deltoid) joints. The Pearson correlation coefficient between the normalized activity ${u}^{(\mathrm{N})}$ and the normalized cocontraction in the wrist (*r* = 0.97), elbow (*r* = 0.95) and the shoulder (*r* = 0.97) were high, and so the analysis was conducted on the normalized activity ${u}^{(\mathrm{N})}$ only.

In the virtual task, the grasp force served as a proxy measure of the arm’s endpoint stiffness magnitude. In a recent study, we showed that the power grasp force is linearly related to the endpoint stiffness magnitude of the arm^16^, and changes in the grasp force resemble the adaptation in the endpoint stiffness when reaching in an unstable force field^22^.

### Degradation of the fixture during real insertion

The real tool with the clip engraves the fixture as it is repeatedly inserted into it. We used a dynamometer fixed to a movable vertical platform to measure the force needed to insert the real tool with the clip into the fixture for 100 repetitions. This was repeated using ten fixtures to estimate how the mean force needed for insertion changed after multiple insertions (Fig. 1i).

### Statistical testing

To detect any differences in the correlates of motor learning between the real and virtual insertion tasks, each normalized variable e.g., the normalized duration ${T}^{(\mathrm{N})}$, was fit using an exponential function

6 $${T}^{(\mathrm{N})}=c+A{e}^{-\lambda (t-1)}$$

where *t* is the insertion trial number and $\left\{c,A,\lambda \right\}$ are parameters of the exponential fit. $c$ is the normalized variable’s convergent value, $A+c$ is its value on the first trial and $\lambda$ is the rate constant. In the virtual task, the training trials were excluded from the fit. A one-way ANOVA was carried out on each fitted variable to examine the effect of the environment.

A significant change in the normalized variable as a function of the trial number was determined by a post-hoc test on the rate constant $\lambda$. A one-sample t-test was applied when $\lambda$ was normally distributed, and when it violated normality as determined by an Anderson–Darling test, a one-sample sign-test was employed.

## Results

The trajectory of the hand in the real and the virtual insertion was relatively straight for all participants (Fig. 1c). Since the movement in the real insertion (10 cm) was shorter than in the virtual task (20 cm) as the workspace of the robot in the real environment was constrained by the size of the tool and the fixture, all variables henceforth are z-normalized for comparison (see “Methods” for normalization, and Supplementary Fig. S1 for plots of the variables prior to normalization).

We first examined how the correlates of motor learning changed as a function of the trial number. The normalized movement duration decreased rapidly in the first few trials, and slowly declined thereafter (Fig. 1d). Each participant’s data was fit using an exponential function (see “Methods”), and three separate one-way ANOVAs with the environment (real or virtual) as the factor revealed no significant effect on the normalized movement duration’s offset (*F*(1,16) = 1.5, *p* = 0.24), the amplitude (*F*(1,16) = 1.8, *p* = 0.20) nor the rate constant (*F*(1,16) = 1.0, *p* = 0.34) parameters (see Table 1 for mean and SEMs of fitted parameters). A one-sample t-test on the rate constant revealed a significant decline in the normalized duration in both the real (*t*(7) = -2.9, *p* = 0.02) and the virtual (*t*(9) = − 2.8, *p* = 0.02) task.

**Table 1:** The normalized movement duration, speed, impulse, stiffness (cocontraction in the real task, grasp force in the virtual task), and the tangential force were all fitted separately for each participant using an exponential function, whose parameters (mean and SEM) are summarized here.

Normalized variable	Offset parameter *c*		Amplitude parameter *A*		Rate constant *λ*	
	Real	Virtual	Real	Virtual	Real	Virtual
Duration	− 2.0 ± 0.7	− 17 ± 10	4.6 ± 0.6	21 ± 10	− 0.5 ± 0.2	− 0.8 ± 0.3
Speed	− 61 ± 20	− 80 ± 30	59 ± 20	79 ± 30	0.1 ± 0.05	0.3 ± 0.2
Impulse	− 120 ± 45	− 38 ± 25	130 ± 45	41 ± 25	− 0.06 ± 0.03	− 0.2 ± 0.1
Stiffness	− 63 ± 24	− 81 ± 42	66 ± 23	83 ± 42	− 0.8 ± 0.5	− 0.3 ± 0.1
Force	− 110 ± 31	32 ± 15	110 ± 31	− 32 ± 15	− 0.05 ± 0.04	0.15 ± 0.08

Next, we calculated the normalized average tangential speed of the hand as a function of the trial number (Fig. 1e). Unlike the rapid decrease in the normalized movement duration in the first few trials, the normalized speed increased steadily. The parameters of the exponential fits on the normalized speed were all comparable between the real and virtual tasks (offset: *F*(1,16) = 0.3, *p* = 0.58; amplitude: *F*(1,16) = 0.3, *p* = 0.57; rate constant: *F*(1,16) = 1.0, *p* = 0.33). One-sample sign-tests on the rate constant were conducted, as they violated normality, which revealed a significant increase in the normalized speed in the real (*p* = 0.008) and the virtual (*p* = 0.02) environments.

We then examined how the impulse, the integral of the force over time after the tool was fully inserted into the fixture, changed as a function of the trial number by fitting it with an exponential function (Fig. 1f). As with the other variables, the three one-way ANOVAs found no significant effect of the environment on the fitted parameters (offset: *F*(1,16) = 3.1, *p* = 0.1; amplitude: *F*(1,16) = 3.0, *p* = 0.1; rate: *F*(1,16) = 1.1, *p* = 0.3). Post-hoc tests on the rate constant using a one-sample sign test detected a decline in the normalized impulse in both the real (*p* = 0.008) and the virtual (*p* = 0.002) tasks.

We then examined how the arm’s endpoint stiffness magnitude changed as a function of trial number (Fig. 1g). In the real insertion task, the endpoint stiffness magnitude was estimated using the average normalized muscle activity in the wrist, elbow and shoulder muscles since the average muscle activity of the arm and its endpoint stiffness are knowingly linearly correlated from previous perturbation studies^15,20^. In the virtual insertion task, the power grasp force was taken as a proxy measure of the endpoint stiffness magnitude since the grasp force and the average normalized activity of the muscles in the arm are linearly related to one another^16^. Since these measures served as a proxy of normalized stiffness magnitude, we analyzed them together. Once again, the exponentially fit parameters were not significantly different between the real and virtual environments (offset parameter: *F*(1,16) = 0.1, *p* = 0.7; amplitude: *F*(1,16) = 0.1, *p* = 0.7; rate constant: *F*(1,16) = 1.2, *p* = 0.3). The normalized stiffness magnitude decreased significantly with the trial number as two one-sample sign tests revealed a significantly negative rate constant in both the real (*p* = 0.008) and the virtual (*p* = 0.002) tasks. To summarize the results thus far, the two groups of participants learning the real and virtual insertion had comparable changes in the normalized trial duration, the speed, the force impulse and the endpoint stiffness magnitude over trials, thereby supporting our hypothesis.

Finally, we examined how the tangential force during insertion changed as a function of the trial number (Fig. 1h). Here, the three one-way ANOVAs revealed a significant difference in the normalized force’s offset (*F*(1,16) = 18.3, *p* < 0.001), the amplitude (*F*(1,16) = 18.9, *p* < 0.001) and the rate constant (*F*(1,16) = 4.6, *p* = 0.047) parameters between the real and virtual environments. Post-hoc tests using two one-sample sign tests revealed a significant decline in the normalized force in the real task (*p* = 0.008) but not in the virtual one (*p* = 0.8), which is explained by the degradation of the real fixture after repeated use. We conducted a control experiment wherein the force needed to insert the task was measured using a dynamometer (see “Methods”). The force needed for insertion declined with repeated use as the clip gradually engraved the fixture (Fig. 1i), thereby reducing the force needed for insertion.

**Figure 2:**
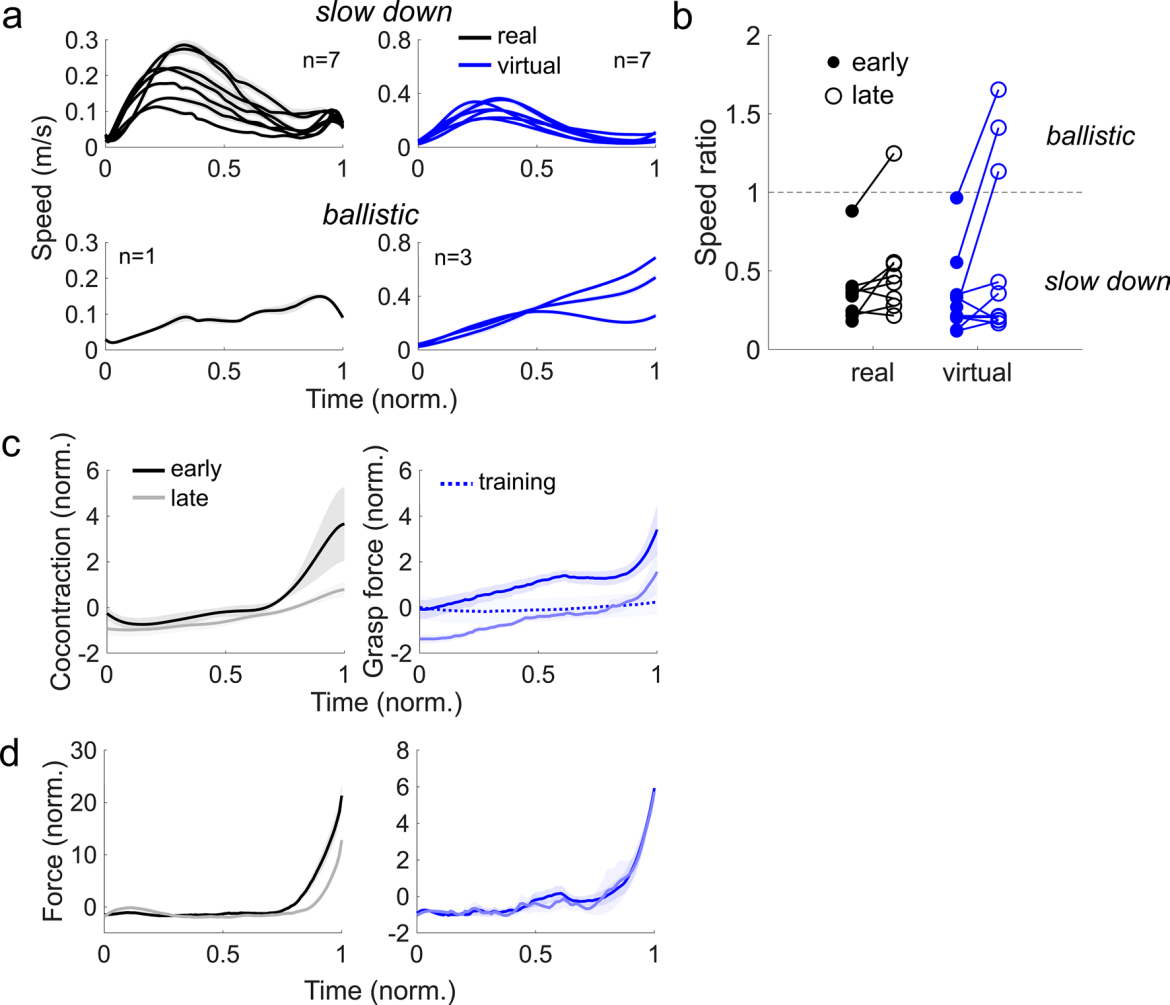
Participants employed either a slow or a ballistic movement when inserting the tool into the fixture. (**a**) Normalized speed time-series averaged in the final phase of the task in the real (black) and virtual insertion (blue). The speed was maximal in the first half of the movement for most participants, who tended to slow down prior to the insertion. Some participants exhibited a ballistic movement where the speed at the end was faster than the first half of the movement. (**b**) Speed ratio in the real and virtual conditions in the early and late stage of the task. Participants exhibiting ballistic movements increased their speed ratio as a function of the trial number, while it remained relatively unchanged for those who slowed down. (**c**) Normalized cocontraction and grasp force as a function of time in the early and late phase. Both increased steadily from the start to the end of the movement, and were lower in the late phase. The grasp force in training trials prior to the insertion task was lower than in the early phase, but higher than the late phase. (**d**) Normalized force as a function of time in the early and late phase of the task. The force was smaller in the late phase in the real task, but remained constant in the virtual task.

The change in the speed, the force and the endpoint stiffness have so far been examined between trials. Since the number of submovements knowingly reduce with motor learning^23^, a change in the speed, force and endpoint stiffness time-series could also occur as a function of the trial number. Upon examining the speed time-series (Fig. 2a), where the time was normalized for comparison, we observed that most participants slowed down at the end of the trial when the insertion took place, while others exhibited a peak speed near the end (Fig. 2a). To delve further, we calculated the speed ratio, defined as the speed at the end of the trial (time of peak tangential force inside the fixture) divided by the peak speed in the first half of the trial duration, for each participant in the early (first 10 trials) and late phase (last 10 trials) of the task (Fig. 2b). Four Anderson–Darling tests revealed that the speed ratio in both environments and in both phases of the experiment violated normality (Holm–Bonferoni corrected significance levels, *p* = 0.02 and *p* = 0.02 in real early and late; *p* = 0.01 and *p* = 0.002 in virtual early and late). Upon inspection, the speed ratios could be classified into two groups. We used k-means clustering (up to three clusters) on the speed ratios of all participants in the late phase of both tasks, and employed the Calinski–Harabasz criterion to identify the optimal number of clusters, which was 2. Adding more clusters only broke apart the existing two clusters. Fourteen participants (n = 7 real, n = 7 virtual) had a speed ratio smaller than 0.5. This *slow down* insertion strategy was characterized by a large peak speed in the first half of the trial, and a low speed at the end. In contrast, four participants (n = 1 real, n = 3 virtual) exhibited a peak speed in the latter half of the movement near the time of insertion. This *ballistic* strategy was characterized by a speed ratio greater than one.

We next looked at the cocontraction and the grasp force time-series, which were smaller in the beginning of the movement (Fig. 2c). To analyze their changes as a function of time, we split the data into the first and final halves of the trial duration, and calculated the mean of the normalized cocontraction and the grasp force in each half of the trial duration as the effect of time. The change as a function of trials was determined by calculating the mean in the first ten and the last ten insertion trials, defined as the early and the late phase of the task. Normalized cocontraction decreased as a function of the trial number (*F*(1,7) = 7.0, *p* = 0.03), but only in the second half of the trial (*p* = 0.05) and not the first (*p* = 0.7). The normalized cocontraction was also greater in the first half of the trial (*F*(1,7) = 7.2, *p* = 0.03), but this was only evident in the early (*p* = 0.01) but not the late (*p* = 0.2) phase of the task. There was no interaction between the two (*F*(1,7) = 2.3, *p* = 0.2).

A similar analysis was conducted on the grasp force in the first and the final half of the trial duration to see its change in time, and to examine their respective changes with the trial number. The time-profile of the grasp force in the early and the late phase of the insertion task was remarkably similar, and only different in their baseline values, almost as if the grasp force was reduced throughout the entire movement over trials (right panel of Fig. 2c). Statistical analysis revealed that the normalized grasp force increased with time (*F*(1,9) = 29, *p* < 0.001) in both the early (*p* < 0.001) and the late phase (*p* < 0.001) of the task. Furthermore, a significant reduction in the grasp force occurred with the trial number (*F*(1,9) = 18, *p* = 0.002) during the first (*p* < 0.001) and the second halves of the trial (*p* < 0.001).

The changes in the time-series of the normalized cocontraction and the normalized grasp force should be considered in the context of how the time-series of the tangential force changed as a function of the trial number (Fig. 2d). While the time-series of the normalized force was comparable between the early and the late phase of the virtual insertion task, the normalized force appeared to be smaller in the final half of the trial at the late phase of the real task in comparison to the early phase. A two-way repeated measures ANOVA on the normalized force in the virtual task confirmed these initial assessments, revealing only a significant influence of the time (*F*(1,9) = 38, *p* < 0.001), and no effect of the trial number (*F*(1,9) = 3, *p* = 0.14) nor of the interaction effect (*F*(1,9) = 2, *p* = 0.2). Post-hoc tests using Tukey’s HSD revealed that the normalized force in the virtual task was greater in the final half of the trial in both the early (*p* < 0.001) and the late phase of the virtual insertion task (*p* < 0.001), but did not change with the trial number. In contrast, a two-way repeated measures ANOVA on the normalized force in the real task revealed a significant effect of the trial number (*F*(1,9) = 9, *p* = 0.02), the time (*F*(1,9) = 57, *p* < 0.001) and the interaction effect (*F*(1,9) = 13, *p* < 0.001). Critically, post-hoc tests revealed that the normalized force in the final half of the movement had decreased in the final phase of the real insertion task (*p* < 0.001). The time-series of the normalized force did not change as a function of the trial number during the virtual environment, but it did decrease in the real task, thereby confirming our second hypothesis.

The decrease in the tangential force when the insertion took place (Fig. 2d) may have been due to the degradation of the fixture (Fig. 1i). Since the fixture did not degrade in the virtual task, the tangential force time-series did not change with the trial number.

## Discussion

We tested a group of participants who inserted a real tool with a clip into a fixture, and a separate group who inserted a virtual tool into a fixture, and compared the changes in the correlates of motor learning. The trial duration, the movement speed, the force impulse and the endpoint stiffness all decreased with the trial number. Importantly, the reduction in these values was similar in both environments, supporting our first hypothesis that the learning of a tooling task in a real and virtual environment are comparable. Furthermore, the force only declined in the real task due to the degradation of the fixture, and remained constant in the virtual task, confirming our second hypothesis.

While the z-normalization of the variables could be used to compare the motor learning in the real and virtual insertion tasks, we should note that the reaching movements were longer in the virtual task (20 cm) relative to the real task (10 cm), and that two different robotic interfaces, albeit with similar endpoint inertia (1.5 kg in the real interface, 1 kg in the virtual one), were used in the study. Furthermore, the endpoint stiffness magnitude was estimated using the arm’s average muscular activity in the real task, while the power grasp force was used in the virtual task. Though these differences do not invalidate our results, they should be interpreted with these differences in mind.

Two insertion strategies were discerned from the time-series of the movement speed. Most participants (14/18 = 78%) resorted to a slow down strategy where the tool was slowed down upon approaching the fixture for insertion, possibly to increase the accuracy of the tool’s position^24^. On the other hand, some participants (4/18 = 22%) used a ballistic strategy where the speed peaked near the end of the trial at the insertion. A prominent theory in motor control suggests that the control of motion and interaction are independent of each other^5,6^. The ballistic strategy can be reproduced through computational modelling using non-linear control theory, but only if the motion and the interaction are optimized at the same time^25,26^, and not separately as in hybrid control theory^27^. Therefore, the CNS may plan and control the tool during insertion by considering the dynamics in both free motion and during interaction together.

Several studies have observed a trial-by-trial decrease in the arm’s cocontraction during motor learning^13,14^, which is attributed to the CNS learning a model of the task’s dynamics, enabling it to use lower endpoint stiffness during the task^28^. We expected a reduction in the cocontraction and the grasp force i.e., the measures of endpoint stiffness magnitude, to take place only during the interaction phase of the insertion. However, both measures of endpoint stiffness magnitude were smaller during the entire movement. The cocontraction time-series at the end of the movement in the real task changed from the early to the late phase, but this reduction may have been due to the decrease in the force needed for insertion as the fixture degraded after repeated use.

This confound was avoided in the virtual task as the force needed for insertion did not change as a function of the trial number as the virtual environment was identical after repeated insertions. Therefore, any change in the grasp force would not be due to a decrease in force. Participants had a constant grasp force time-series during training but increased it in the early phase, foremost at the end of the movement where interaction took place. The grasp force was overall lower in the late phase in comparison to the early phase, but resembled one another in the way they increased and peaked at the end of the insertion. Thus, the CNS needed only a few trials to adapt the time-series of the arm’s endpoint stiffness magnitude. Over time, the endpoint stiffness magnitude decreased throughout the entire movement, and not at specific intervals.

The correlates of motor learning, namely the trial duration, the movement speed, the impulse and the endpoint stiffness magnitude, adapted in a comparable manner in a real and virtual insertion task. Furthermore, the two insertion strategies discerned in the real task were observed in the virtual task too. A previous study^9^ reported that the movement duration and the tangential force were greater in the virtual task. Unfortunately, such direct comparisons are not possible in our study as the movement lengths were different (10 cm in the real versus 20 cm in the virtual task) and the simulated fixture was different from the real one. Thus, our study is constrained to examining the sequence of motor learning over repeated trials.

Importantly, the dynamics of the task was controlled precisely in a virtual environment, unlike the real insertion task where the degradation of the fixture changed the insertion dynamics of the task. Altogether, a virtual environment with haptic feedback of the dynamic interaction could be used to analyze the motor learning during tool use. However, the real world is replete with non-linear dynamics and physical interaction that are challenging to implement in virtual environments. As such, more evidence is necessary comparing the learning of a task in a virtual and real environment. Further studies could analyze how the learning of a virtual tooling task transfers to a real one.

If the environment is virtual, various methods can be used to probe motor learning and motor memory in the literature e.g., catch trials where the haptic feedback is suddenly eliminated, to further our understanding of motion planning in tooling tasks. The versatility of the virtual environment provides the freedom to change the dimensions of the tool or the fixture to make the task more or less challenging depending on the user’s performance, which may be useful in training new recruits or polishing the skills of an experienced user.

## Supplementary Information

Supplementary Figure S1.
