# Supplementary material for: Analogous adaptations in speed, impulse and endpoint stiffness when learning a real and virtual insertion task with haptic feedback
Source: Sci Rep. 2020 Dec 18;10:22342. doi: 10.1038/s41598-020-79433-5 (PMC7749137; doi:10.1038/s41598-020-79433-5)
Supplement: Supplementary file 1 — Supplementary Figure S1. [file 41598_2020_79433_MOESM1_ESM.docx]

**Supplementary Information to ‘Analogous adaptations in speed, impulse and endpoint stiffness when learning a real and virtual insertion task with haptic feedback’**

Atsushi Takagi^1,2,3^*^†^, Giovanni De Magistris^4,^*, Geyun Xiong^2^, Alain Micaelli^4^, Hiroyuki Kambara^2^, Yasuharu Koike^2^, Jonathan Savin^5^, Jacques Marsot^5^ and Etienne Burdet^6^

^1^NTT Communication Science Laboratories, 3‐1 Morinosato Wakamiya, Atsugi, Kanagawa, 243-0198, Japan.

^2^Tokyo Institute of Technology, 4259 Nagatsuta-cho, Yokohama 226-8503, Japan

^3^Precursory Research for Embryonic Science and Technology (PRESTO), Japan Science and Technology Agency (JST), 4-1-8 Honcho, Kawaguchi, Saitama 332-0012, Japan.

^4^ CEA, LIST, LSI, rue de Noetzlin, Gif-sur-Yvette, F-91190, France

^5^Institut National de Recherche et de Sécurité (INRS), rue du Morvan, CS 60027, Vandoeuvre-lès-Nancy, F-54519, France.

^6^Imperial College of Science, Technology and Medicine, South Kensington, London SW7 2AZ, UK.

*equally contributing authors

^†^atsushi.takagi.yx@hco.ntt.co.jp

*
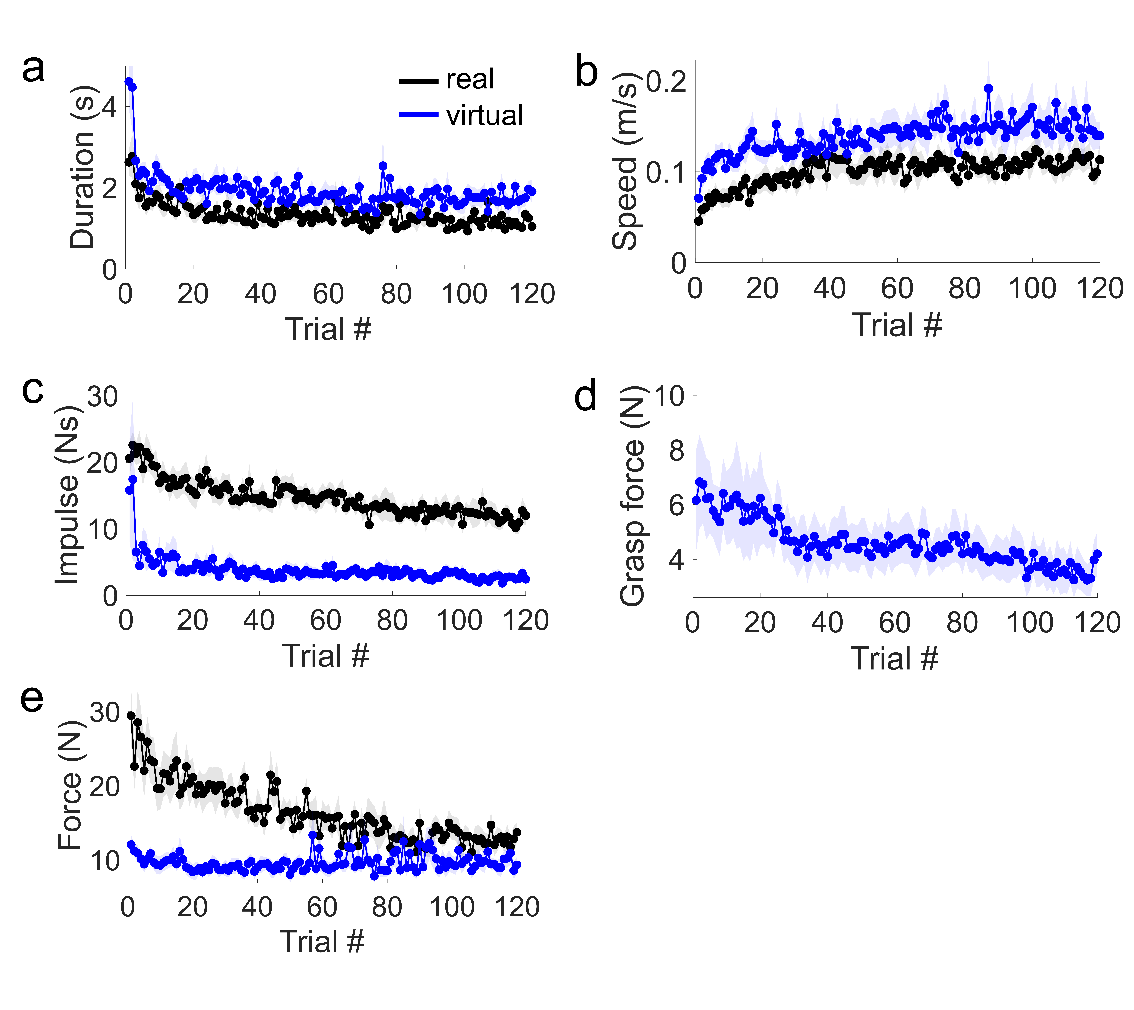
*

**Supplementary Figure S1.** The actual values of the movement duration, speed, impulse, grasp force and the tangential insertion force in the real and virtual tasks (raw versions of the normalized Figures 1d-h). The plot of cocontraction is omitted as the raw EMG must be normalized to extract a meaningful group mean value.
